# A Decade-Long Cohort Analysis of Human Cytomegalovirus (HCMV)-Induced Early and Late Renal Rejection in Post-Transplant Patients in the Eastern Indian Population

**DOI:** 10.3390/v16060847

**Published:** 2024-05-25

**Authors:** Debsopan Roy, Aroni Chatterjee, Atanu Pal, Rajendra Prasad Chatterjee, Nilanjan Chakraborty

**Affiliations:** 1 Virus Research Laboratory, ICMR-National Institute of Cholera and Enteric Disease, Kolkata 700010, West Bengal, India; 2 Department of Biotechnology, School of Biotechnology and Bioscience, Brainware University, Kolkata 700125, West Bengal, India; 3 Department of Nephrology, IPGME&R-SSKM, Kolkata 700020, West Bengal, India

**Keywords:** human cytomegalovirus, renal rejection, latent infection, clinical outcome

## Abstract

**Background**: HCMV causes severe clinical complications in transplant recipients and may lead to graft rejection. Successful renal transplantation heavily relies on the early prevention and diagnosis of CMV infections, followed by prompt prophylactic treatment before transplantation. Despite the majority of renal rejection cases with acute HCMV infections being asymptomatic and occurring one to two years later, the objective of this research was to comprehend the effect of late HCMV infection on renal rejection by examining specific clinical parameters in the Eastern Indian cohort. **Method**: In this study, 240 patients were studied for five years following transplantation, and their data were collected from the local metropolitan hospital in Eastern India. Both HCMV-positive and -negative post-transplant patients were investigated using the clinical parameters and viral loads for latent infection. **Results**: Within the studied population, 79 post-transplant patients were found to be HCMV positive. Among them, 13 (16.45%) patients suffered from renal rejection within less than 2 yrs. of transplantation (early rejection) and 22 (27.84%) patients suffered from renal rejection after 2 yrs. from the operation date (late rejection). Assessment of clinical parameters with respect to HCMV infection revealed that in early rejection cases, fever (*p*-0.035) and urinary tract infection (*p*-0.017) were prominent, but in late rejection, hematuria (*p*-0.032), diabetes (*p*-0.005), and creatinine level changes (*p* < 0.001) were significant along with urinary tract infection (*p*-0.047). **Conclusions**: This study provides valuable insights into monitoring latent CMV infections and highlights the understanding of reducing renal rejection rates and the need for further research in this field.

## 1. Introduction

Human cytomegalovirus (HCMV) is a member of the Herpesviridae family, and the subfamily Beta-herpesviridae consists of double-stranded DNA and can cause end-organ diseases (EODs) in 20 to 60% of transplant recipients. HCMV is a significant cause of increased morbidity and mortality in this population, as well as graft rejection [1]. The direct effects of an HCMV infection within transplant patients can lead to either acute or chronic infectious disease syndromes. These may present as symptoms such as fever, leukopenia, and hepatitis, among others. The outcome of these direct effects depends on the past exposure and experience of both the transplant donor and the recipient with the virus, as well as the immunosuppressive treatment regimen used [2]. One of the important factors that determines the risk of post-transplantation HCMV complications is the serostatus of both the donor (D) and the recipient (R) prior to transplantation. Studies have shown that individuals in the D+/R− (only donor-positive) and D+/R+ (both donor- and recipient-positive) groups are at a higher risk of HCMV-related complications after transplantation [3,4]. Therefore, monitoring the serostatus of donors and recipients can help reduce the incidence of complications and improve the overall outcome of transplantation [5]. HCMV infection after transplantation can cause damage to the vasculature, leading to a range of complications, including rejection of a renal allograft, atherosclerosis, and thrombotic microangiopathy [6]. Additionally, it can result in bone marrow suppression, respiratory and gastrointestinal ailments, and neurological complications [7,8,9]. Hence, monitoring for CMV infection post transplantation is crucial to prevent potential complications and improve patient outcomes. In the current medical landscape, the effectiveness of renal transplantation is heavily reliant on the ability to prevent or diagnose CMV infections early on and administer timely prophylactic treatment prior to transplantation [10]. However, there is a significant lack of research on the occurrence of late-onset HCMV infection and its correlation with post-transplant renal rejection. Effective management and vigilant monitoring of HCMV infectivity following transplantation are crucial. Research has shown that acute HCMV infection can result in renal rejection within one year of transplantation [11], but the majority of cases occur asymptomatically 1–2 years later. It is therefore imperative to remain vigilant and proactive in the ongoing management of HCMV infectivity.

In this article, over the course of a decade-long study (2010–2023), a cohort of 240 individuals who received a renal transplant and were admitted to the Department of Nephrology at SSKM Hospital in Kolkata, India, was examined. The study aimed to gain a deeper understanding of the impact of late HCMV infection on renal rejection by analyzing specific clinical parameters.

## 2. Materials and Methods

### 2.1. Patient Selection

This study analyzed the presence and effects of CMV infection after transplantation, with a follow-up period of up to 3 years. This study involved a sample of 252 patients who had undergone renal transplants and were infected with HCMV. It was conducted at a single center, and we used a combination of retrospective and prospective methods. The study monitored the patient’s previous HCMV serostatus and other clinical parameters, and we documented the impact of the post-transplant scenario and conducted follow-up investigations to understand the correlation between HCMV infection and renal rejection. Among the 252 patients, 12 patients were excluded due to discontinued follow-up (9 patients) or other health complications resulting in death (3 patients) within one year of transplant. The remaining 240 patients underwent HCMV-antigenemia (Ag) and quantitative DNA PCR testing at 6-month intervals over the 5-year period after transplantation. All sample collection procedures were approved by the institutional ethics committee in accordance with the 1964 Helsinki Declaration, and the study itself was approved by the institutional Ethics Committee of the IPGME&R, Kolkata, in collaboration with the Department of Nephrology, IPGME&R, Kolkata (Inst.IEC/935-11.10.2010 and IPGME&R/IEC/2022/283-30.06.2022). All of the clinical signs and symptoms of both HCMV-positive and -negative patients after the transplant were recorded in each follow-up observation. Routine serum biochemical reports were also documented for further analysis. Renal rejected patients were selected initially on the basis of ultrasound-guided real-time percutaneous needle biopsy. In this process, two cores were taken for the study. One was for light microscopy to identify the details of the pathology, particularly T-cell-mediated rejection (TCMR), and the other one was for immunofluorescence with antibody-mediated rejection (ABMR), which was performed on both C4d-positive and -negative patients.

### 2.2. Sample Collection

Peripheral blood samples were collected in EDTA-containing tubes (5–10 mL) and processed through centrifugation (1000× *g* for 10 min), and the serum was frozen at −80 °C for future use.

#### 2.2.1. HCMV Infection Determination Using HCMV-Antigenemia Assay

The HCMV infection status of patients was assessed using the Millipore HCMV pp65 Antigenemia-3247x assay (HCMV-Ag), which measured the presence of the lower matrix protein pp65 in peripheral blood leukocytes. Samples were collected within 6 h of collection and tested according to the manufacturer’s instructions. A positive HCMV-Ag result was determined through the detection of at least one HCMV-positive cell per 250,000 leukocytes. Patients who tested positive were regularly monitored on a weekly basis during the first month of antiviral treatment post-transplant and then every 3 months for up to 2 years (chronic HCMV infection phase). After 1 year, the HCMV-Ag test was monitored in every positive patient in 3-month intervals until HCMV-Ag-positive lymphocytes were observed below the cut-off value (no HCMV-positive cells). For negative patients, CMV-Ag was monitored at the same interval during follow-up.

Post-transplant patients with HCMV infections were treated with orally administrated ganciclovir with a dosage of 500–750 mg/kg/day as per the patient’s clinical conditions and creatinine clearance (ranging from 30–72 mL/min) for 20–25 days. The first HCMV-Ag test was initiated 18–20 days after transplantation for both the donor and recipient. The dosage was adjusted accordingly as per the follow-up HCMV-Ag report and continued until the HCMV-Ag was below the cut-off range. Similarly, prior to transplantation, all recipients, as well as respective living donors, were subjected to ganciclovir prophylaxis with a dosage of 500 mg/kg/day for 15 days as per clinicians’ observations. The dosage of ganciclovir varied among the patients who had undergone both early and late rejection followed by positive HCMV-Ag as well as clinical symptoms [12].

#### 2.2.2. HCMV Viral Load Determination and Quantification of Major Virion Tegument Protein and Latency-Associated Genes

To extract the entire DNA from each blood serum sample, the QIamp DNA blood Mini Kit from Qiagen Inc., Germantown, MD, USA (Cat-51106) was used. To quantify HCMV infection using a major HCMV early immune evasion tegument protein, qualitative PCR was performed using the UL83 gene [13], as well as the major HCMV glycoprotein (B) gB, which is responsible for viral entry [14]. We utilized the Primer 3 online tool to design these primers. For quantifying viral latency, we utilized the HCMV latency-related US-28 gene. The forward and reverse primers for UL-83 were 5′-GGG ACA CAA CAC CGT AAA GC-3′ and 5′-GTC AGC GTT CGT GTTTCCCA-3′, respectively. For UL-55 (gB), the primers were 5′GGTCTTCAAGGAACTCAGCAAGA-3′ and 5′-CGGCAATCGGTTTGTTGTAAA-3′, while for US-28, they were 5′-TTACGTTGTTTCTGTACGGC-3′ and 5′-AGACAGTGTAGTTCTTGCGA-3′. For quantitative estimation of HCMV gene products (UL-83, UL-55, and US-28) total RNA was extracted using TRIzol reagent (Invitrogen, Waltham, MA, USA; Thermo Fisher Scientific, Inc., Waltham, MA, USA). The normal approach was followed to convert RNA into cDNA at 42 C for 60 min using the Primescript 1st strand cDNA synthesis kit from TAKARA. cDNA was amplified in a standard real-time PCR experiment using SYBR green dye (TB Green premix ex taq, TAKARA, Shiga, Japan). The reaction mixture involving 25 μL of total PCR reaction mixture included with 12.5 μL of 2× TB Green premix (TAKARA), 1.5 μL of each forward and reverse primer (10 pmol/μlConc.), 7.5 μL sterile water, and 2 μL of cDNA samples were used. The thermal cycler was set to be performed at 95 °C for 5 min for early denaturation, 95 °C for 45 s, 57.5 °C for 30 s, 72 °C for 45 s for 35 cycle repeats, and 72 °C for 5 min for final extension. The cDNA input amount was standardized using internal control GAPDH transcripts via real-time PCR. Real-time expression ratios of mRNAs were determined using the 2^−ΔΔCt^ method for relative quantification.

### 2.3. Statistical Analysis

SPSS version 25.0 (IBM Corp. Released 2017. IBM SPSS Statistics for Windows, Version 25.0. Armonk, NY, USA: IBM Corp.) was utilized for statistical analysis. In order to test the continuous variables between the means of the study groups, either Student’s *t*-test or Welch’s test was used. For categorical variables, Chi-squared and Fischer’s exact tests were conducted on the groups. To perform multivariate analysis, a multiple logistic regression model was used. Effects were deemed significant if *p* < 0.05.

## 3. Results

### 3.1. Demographic Parameters of the Patient Samples

In this study, patients were divided based on their positive or negative HCMV-Ag and qualitative PCR assay results after transplantation. Of the 240 patient samples, 182 were male and 58 were female. The HCMV-positive samples accounted for 32.91% (79/240) of the total, while negative samples accounted for 67.08% (161/240). The median age range of both groups was 46–53 years and no significant differences were observed between the two groups (*p*-0.894). Samples were collected from both rural and urban areas, with no significant difference observed in HCMV infectivity between the two (*p*-0.356). Follow-up investigations were conducted for a median range of 60–66 months, with positive cases being strictly observed for at least 62 months. Both living and deceased donors were selected for transplantation among the 240 samples. Of the 187 living donors, 65 were HCMV seropositive (34.75%) and 122 were seronegative (65.24%). Similarly, of the 53 deceased donor samples, 14 were HCMV seropositive (26.41%) and 39 were HCMV seronegative (73.58%). No significant difference in HCMV status was observed between ABO-compatible and non-compatible samples (*p*-1.000). The study included a total of 240 patients, 93 of whom received organs from family members, while the remaining 147 received organs from non-related donors. Among the related donors, 26 out of 79 (32.91%) were found to be HCMV seropositive, while 34 out of 161 (21.11%) were seronegative. In contrast, among non-related donors, 53 out of 79 (67.08%) were HCMV positive, and 92 out of 161 (57.14%) were seronegative. Based on tissue compatibility and HCMV serostatus before transplantation, both donors and recipients were divided into four distinctive groups: D+R+, D+R−, D−R+, and D−R−. After transplantation, patients in the D+R+ (31.64%), D+R− (35.44%), and D−R+ (32.91%) groups were found to be HCMV seropositive and were therefore subjected to the HCMV therapeutic strategy after the operation. A noteworthy finding pertained to the correlation between HCMV infection and renal rejection status in post-transplant patients, regardless of whether the transplant occurred within the past two years of the transplant or beyond. Out of the 79 cases of HCMV infection, a total of 22 patients were rejected within two years of their transplant and 13 patients among them experienced HCMV infection (*p*-0.004); while 22 of the 33 patients who experienced renal rejection beyond two years were HCMV positive (*p* < 0.001). Patients who experienced renal rejection were identified through both antibody-mediated histopathology and tissue biopsy. It was observed that out of the thirteen patients who were seropositive for HCMV within 2 years of transplantation, seven showed positive antibody responses, while six were confirmed to have experienced rejection through biopsy. Similarly, among the 22 patients who experienced late renal rejection (more than two years after transplantation), tissue biopsy was found to be a significantly more accurate diagnosis method compared to antibody-mediated histopathology, with 20 patients being correctly diagnosed through biopsy (*p* = 0.027) (Table 1).

### 3.2. Assessment of Clinical Risk Factors for HCMV-Positive Early Renal Rejected Patients (<2 Years)

A comparison of clinical and biochemical parameters was conducted between HCMV-positive renal rejected and non-rejected patients within two years of transplant. As most cases had organ donors who were HCMV-positive (due to D+R+ and D+R− groups), the relationship between positive donors and renal rejection was also assessed. HCMV-associated clinical manifestations were observed among patients who underwent renal transplants and experienced rejection. However, there were no significant differences observed between HCMV asymptomatic and symptomatic manifestations. All patients were given HCMV prophylaxis prior to the transplant. However, due to diverse seropositive donor (D+/−) and recipient (R+/−) groups, many patients were found to have reoccurring HCMV infections.. There was no correlation found between pre-transplant HCMV seropositive donors and recipients and post-transplant HCMV positivity in the renal rejected patients. Among the clinical symptomatic parameters, renal rejected patients were found to have significantly higher fever (*p*-value 0.017) and urinary tract infections (*p*-value 0.007) rates compared to non-renal rejected HCMV patients. Other clinical parameters such as hematuria, leukopenia, diabetes, and diarrhea were found to be insignificant between these two groups. Serum biochemical parameters like SGPT, ALP, and creatinine were also found to be insignificant in early rejection cases among these groups (Table 2).

### 3.3. Analysis of Specific Clinical Parameters in HCMV-Associated Late Renal Rejection Patients (>2 Years)

An analysis of HCMV-related renal rejection cases was conducted to assess clinical manifestations and correlations between pre-transplanted positive donors/recipients and persistent HCMV-infected post-transplant rejections. In the univariate analysis, a correlation was found between asymptomatic patients with HCMV and late renal rejection cases (*p* = 0.040). However, in the multivariate analysis, this correlation was deemed insignificant. Similarly, the univariate test showed that the recurrence of HCMV infection among late renal rejected patients due to pre-transplanted HCMV-positive recipients was significant (0.011). This association, however, also became insignificant in the multivariate analysis. However, certain clinical parameters were observed to be significantly changed in late rejection cases. Hematuria was found to be significantly correlated (*p*-0.032), as well as persistent urinary tract infection as a significant risk factor (*p*-0.047). Additionally, diabetes incidence was significant (*p*-0.005) compared to non-rejected HCMV-positive patients. Although Leukopenia was initially observed to be a serious risk factor, multivariate analysis reported this parameter to be insignificant. In terms of biochemical parameters, a high level of creatinine was found to be significant (*p* < 0.001) among the late renal rejected positive patients compared to non-rejected HCMV positive groups (Table 3).

### 3.4. Comparison of Early and Late Renal Rejection in Relation to HCMV Infectivity

Regarding the HCMV-Ag assay, it was found that the number of HCMV-positive cells (mean ± SD) was significantly higher (~9 ± 2 cells) in renal rejected patients during early post-transplant rejection compared to non-rejected patients (~6 ± 2 cells) (*p*-value 0.030). However, in cases of late renal rejection, the number of positive HCMV cells in leukocytes was reduced compared to early rejection cases, with no significant variations observed between the two groups (*p*-value 0.224). When analyzing HCMV viral load, it was observed that renal rejected patients during early infection stages had a significantly higher viral load (7.84 + 0.89 DNA log copies) compared to non-rejected patients (6.34 + 1.08 DNA log copies) (*p*-value 0.007). Similarly, in late rejection cases, the viral log copy number was reduced in both groups, but a significant variation was still observed with higher HCMV viral loads in renal rejected patients (*p* < 0.001). Quantitative estimation of CD-4+ cells was also measured among both early and late renal rejected patients. In early cases, no significant variations were observed (*p*-0.384), but in later rejected cases with persistent HCMV infection, CD-4+ counts were significantly reduced (*p* < 0.001) compared to non-rejected patients. Among the biochemical parameters, creatinine level in early rejection cases was found to be insignificant among the groups (*p*-0.331), but a significant elevation in creatinine (2.62 + 0.81 ng/dL) was observed in the late renal rejected group (*p* < 0.001) (Table 4).

**Figure 1:**
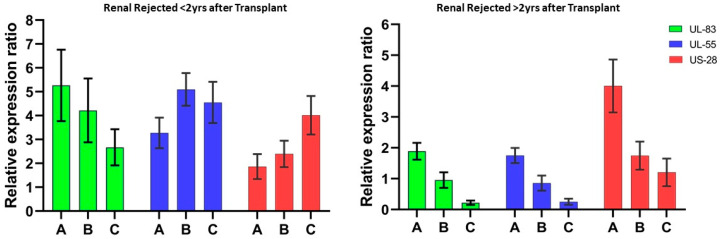
Relative expression ratio of HCMV genes after transplantation. The quantitative expression of the HCMV early immune evasion gene (UL-83), major HCMV structural glycoprotein genes (UL-55), and latency-associated gene (US-28) were analyzed during initial as well as follow-up studies. (A) Initial observation after transplantation; follow-up observation at (B) 3 months and (C) 6 months.

Relative mRNA expression of HCMV early immune evasion tegument protein and latency-associated genes showed very distinctive expression profiling in both early renal rejected (<2 years) and late renal rejected patients (>2 years). The study was conducted during the initial observation of renal rejection confirmatory tests (antibody-mediated or tissue biopsy) and was followed up in the initial and 3-month intervals up to 6 months. It has been observed that during early rejection cases, UL-83 (expression ratios of 5.26 and 3.27) and UL-55 genes (expression ratios of 4.21 and 5.09) were comparatively high (*p* < 0.001) compared to HCMV latency-related gene US-28 (expression ratios of 1.86 and 2.39), whereas in late renal rejected cases it was found that the relative expression of US-28 (ratios of 4.02 and 1.76) at the initial observation was significantly higher (*p* < 0.001) in comparison with HCMV genes UL-83 and UL-55 (expression ratios of 1.39 and 0.95 and 1.75 and 0.85, respectively) (Figure 1).

### 3.5. Comparison of Renal Rejection with HCMV-Related and Non-Related Patient Groups

A comparative analysis was conducted among renal rejected patients with and without HCMV infection to determine the impact of various risk factors. The analysis focused on the univariate comparison of different parameters associated with the patient’s conditions. The results revealed that several factors played a significant role in HCMV-induced renal rejection. Hematuria, leukopenia, diabetes, and urinary tract infection (UTI) were identified as the major parameters that had a strong correlation with the condition (*p* < 0.001). The study investigated the biochemical parameters involved in renal rejection cases induced by human cytomegalovirus (HCMV) infection. It was found that creatinine levels were expressed in the majority of both HCMV-induced and non-induced renal rejection cases, with them being a major biochemical marker for renal rejection and indicating impaired kidney function. Although other biochemical markers like alkaline phosphatase and SGPT were slightly high in HCMV-infected renal rejected patients, the difference was statistically insignificant. However, the CD-4+ cell count, which plays a crucial role in the body’s immune response, was significantly reduced in HCMV-infected renal rejected patients (*p* = 0.031). This finding suggests that HCMV infection may compromise the immune system and thus increase the risk of renal rejection (Table 5).

## 4. Discussion

Renal rejection manifests in two distinct forms: acute renal rejection and chronic renal rejection. Acute transplant rejection can arise within days to months following a transplant when the immune system perceives the new organ as foreign and mounts an attack. Although acute transplant rejection is prevalent, the prognosis is uncertain [15]. It has been reported that kidney transplant patients commonly suffer from cytomegalovirus (HCMV) infection, which can increase morbidity and decrease graft survival, resulting in acute renal rejection [16] Chronic renal rejection occurs slowly over years after a kidney transplant when the newly transplanted kidney stops functioning due to constant attacks from the host immune system [17] According to a previous study, the role of human cytomegalovirus (HCMV) in the pathogenesis of chronic rejection is contingent upon the presence of acute rejection. There exist two potential reasons for acute rejection. Firstly, the presence of HCMV disease may hinder adequate treatment. Secondly, recipients undergoing treatment for acute rejection may experience increased virulence of the latent HCMV virus [18]. Previous research has shown that dormant HCMV poses a distinct risk to the longevity of transplanted kidneys. It has been identified as an independent factor contributing to graft failure, with a greater likelihood of failure than mortality. These results reinforce the idea that latent HCMV can have detrimental effects on transplanted organs [19]. In our cohort analysis, we tried to decipher the comparison between early renal rejection due to chronic HCMV infection and late rejection due to latent HCMV infection in the Indian sub-population. From our overall understanding, it was found that during early renal rejection (<2 yr.), high HCMV viremia was able to drastically regulate our study cohort with no observable symptoms other than urinary tract infection. A recent investigation reported that HCMV viremia >500 DNA copies/µg is sufficient to cause severe health complications as well as renal rejection [20]. It has been observed that administering prophylactic treatment of HCMV drugs to both HCMV-positive living donors and recipients prior to transplantation may not always prevent HCMV recurrence in post-transplant cases. This suggests that there may be additional factors at play that can lead to HCMV recurrence even in previously treated cases. The findings of this analysis highlight the need for further research to better understand the mechanisms underlying HCMV recurrence in transplant cases and to develop more effective strategies to prevent and manage this complication. In instances where renal rejection and HCMV infection are suspected, a combination of antibody-mediated histopathology and tissue biopsy may be employed for early rejection diagnosis. However, in cases where HCMV infection persists for an extended period post transplantation, tissue biopsy has been found to be appreciably superior in affirming the presence of renal rejection. This is due to the fact that tissue biopsy affords a more comprehensive and accurate analysis of the state of the kidney tissue, allowing for the identification of any underlying causes of rejection. As such, in persistently HCMV-infected late post-transplant cases, tissue biopsy is deemed the preferred method for confirming renal rejection. In our cohort analysis, it was found that in early rejection cases, the HCMV viral load was 7.84 ± 0.89 log copies (~5600–7850 DNA copies/µg), which was significantly higher in comparison to non-rejected patients. In the late rejection cases, patients were also initially found to be within similar ranges of DNA viremia but after antiviral therapy, viremia was reduced compared to the early stages, but a gradual significant elevation () was observed within the late renal rejected patients (>2 years) with 2.96 ± 0.94 log copies (~1670–3809 DNA copies/µg) at the time of rejection. HCMV pp65 antigenemia assay is the widely used process to monitor HCMV disease progression in different patient groups [21]. During the early renal rejection cases, the HCMV pp65 antigenemia reports were significantly high in comparison to non-rejected patients, which distinctively supported our observational evidence of HCMV viremia among these groups. The antigenemia assay was carried out throughout antiviral therapy and beyond, as described in Section 2, until no HCMV-infected leukocytes were observed. In this study, as we continued the quantitative estimations of HCMV viral copies using HCMV latency-associated gene US-28 [22] even after obtaining no HCMV-positive leukocytes, it was evident that latent HCMV infection might be playing a critical role in late renal rejection; until present, there is no exact cut-off value for when HCMV latent viremia is determined; thus, in this study when viremia became >500 copies/µg, antiviral therapy was reinitiated. Within this reinitiated therapy, 22/79 patients became non-responsive to treatment and gradual elevation in HCMV viral load (2.96 ± 0.94 log copies) led to renal rejection. Among the 22 late renal rejected patients with latent HCMV infection, 9 patients died before further transplantation, and the remaining 13 patients underwent transplantation again after a prolonged dialysis period and anti-HCMV multi-drug combinational therapy, but only 7 out of 13 were successfully transplanted as well as surviving and 6 patients died due to several other health complications.

The early immune evasion gene (UL-83), major glycoprotein (UL-55), and latency-related gene (US-28) of human cytomegalovirus (HCMV) were analyzed comparatively to understand their expression patterns. The study revealed an intriguing outcome, as significantly higher expression ratios of UL-83 and UL-55 genes were observed in comparison to the US-28 gene in early HCMV-related renal rejection cases. These observations were made during the initial and follow-up stages. It was also observed that in cases of late renal rejection, the relative expression ratio of the US-28 gene was substantially higher than that of the UL-83 and UL-55 genes. This suggests that latent HCMV (human cytomegalovirus) infection, which can persist for over two years post transplantation, may significantly contribute to increasing the incidence of renal rejection. Previous research has shown that a decrease in CD4+ cell counts (<500 cells/mm^3^) was observed in conjunction with active cytomegaloviral infection [23]. Similarly, continuous elevation in creatinine was also found to be a critical risk factor for renal rejection [24]. In our observation, it was found that during the early renal rejection cases, no significant decrease in CD4+ count and elevation in serum creatinine was observed, but in the late rejection cases, CD4+ counts were found to be significantly reduced (mean ~460 cells/mm^3^) as well as the creatinine concentration being significantly elevated to 2.62 ± 0.81 mg/dL compared to non-rejected patients with 1.26 ± 0.67 mg/dL. These results suggest that as CD4+ decreases slightly during latent infection from the normal ranges, the gradual elevation in creatinine concentration might be a significant risk factor along with HCMV viremia for late renal rejection. When considering the clinical risk factors associated with latent HCMV-induced renal rejection, it was found that in both early and late renal rejection cases, urinary tract infections (UTI) were persistent, whereas hematuria and diabetes gradually became predominately significant in later cases.

In summary, our study is the first initial exploration of the occurrence of HCMV-induced post transplantation over a 5-year period across the Indian subcontinent. This investigation also signifies that with continued monitoring of latent CMV infections, we may be able to mitigate the incidence of renal rejection. Nonetheless, additional research is required to determine immune modulation in renal rejected cases with latent HCMV infection to develop new antiviral strategies to reduce graft loss.

## Figures and Tables

**Table 1:** Univariate comparison of demographic characters between the patient groups with or without positive HCMV infectivity.

		CMV Status		*p*-Value
	Total Sample (*n* = 240)	Positive (*n* = 79)	Negative (*n* = 161)	
**Study population**				
Male	182 (75.83%)	49 (62.02%)	133 (82.60%)	
Female	58 (24.16%)	30 (37.97%)	28 (17.39%)	
Median Age (years)	49 (46–53)	48 (46–51)	49 (47–53)	0.894
**Geographic location**				0.356
Rural	113 (47.08%)	34 (43.03%)	79 (49.08%)	
Urban	127 (52.91%)	45 (56.96%)	82 (50.93%)	
**Follow-up time (months)**	63 (60–66)	64 (66–61)	62 (60–63)	
**Donor type**				0.244
**Living**	187 (77.91%)	65 (34.75%)	122 (65.24%)	
**Deceased**	53 (22.08%)	14 (26.41%)	39 (73.58%)	
**Recipient type**				0.157
**Related donor**	93 (38.75%)	26 (32.91%)	67 (41.61%)	
**Non-related donor**	147 (61.25%)	53 (67.08%)	92 (57.14%)	
**Serological status of HCMV-IgG before transplant**				0.164
D+R+	59 (24.58%)	25 (31.64%)	34 (21.11%)	
D+R−	79 (32.91%)	28 (35.44%)	51 (31.67%)	
D−R+	64 (26.66%)	26 (32.91%)	38 (23.60%)	
D−R−	38 (15.83%)	0 (0%)	38 (23.60%)	
**Blood group status (ABO)**				1.000
compatibility	135 (56.25%)	44 (55.69%)	91 (56.31%)	
In-compatibility	105 (43.75%)	35 (44.30%)	70 (43.47%)	
**Renal rejection tested <2 years**				1.000
Antibody mediated (+)	12 (5%)	7 (8.86%)	5 (3.10%)	
Antibody mediated (−)	10 (4.16%)	6 (7.59%)	4 (2.48%)	
**Biopsy tested**	10 (4.16%)	6 (7.59%)	4 (2.48%)	
**Renal rejection tested >2 years**				0.027
Antibody mediated (+)	7 (2.91%)	2 (2.53%)	5 (3.10%)	
Antibody mediated (−)	26 (10.83%)	20 (25.31%)	6 (3.72%)	
**Biopsy tested**	26 (10.83%)	20 (25.31%)	6 (3.72%)	
**Renal rejection**				
Less than 2 years	22 (9.16%)	13(16.45%)	9 (5.59%)	0.004
More than 2 years	33(13.75%)	22 (27.84%)	11 (6.83%)	<0.001

**Table 2:** Regression analysis of HCMV-positive renal rejected patients (<2 years of transplant) in comparison with different risk factors.

	Renal Rejection of HCMV-Positive Samples Less than Two Years (*n* = 13)					
	Univariate Test			Multivariate Test		
Risk Factors	Odds Ratio	95% CI (Lower Upper)	*p*-Value	Odds Ratio	95% CI (Lower Upper)	*p*-Value
**HCMV clinical manifestation transplant**						
Symptomatic	1.93	0.81–3.71	0.457			
Asymptomatic	0.54	0.24–1.18	0.121			
**Reoccurring HCMV infections after HCMV prophylaxis**						
Donor positive	1.12	0.72–1.74	0.689			
Recipient positive	1.03	0.53–1.97	1.000			
**Symptoms**						
**Fever**	2.88	1.01–8.21	**0.017**	7.81	1.16–52.56	**0.035**
**Pneumonia**	0.63	0.30–1.30	0.213			
**Hematuria**	0.96	0.44–2.10	0.925			
**Leukopenia**	0.72	0.30–1.73	0.470			
**Diabetes**	0.86	0.33–2.23	0.768			
**Diarrhea**	1.98	0.81–4.86	0.095			
**UTI**	3.13	1.11–8.79	**0.007**	9.97	1.50–56.21	**0.017**
**Biochemical parameters**						
**SGPT**	1.08	0.51–2.21	0.833			
**Alkaline phosphatase**	0.94	0.56–1.74	0.981			
**Creatinine level**	1.23	0.67–2.25	0.470			

**Table 3:** Regression analysis of HCMV-positive renal rejected patients (>2 years of transplant) in comparison to different risk factors.

	Renal Rejection of HCMV-Positive Samples More than Two Years (*n* = 22)					
	Univariate Test			Multivariate Test		
Risk Factors	Odds Ratio	95% CI (Lower Upper)	*p*-Value	Odds Ratio	95% CI (Lower Upper)	*p*-Value
**HCMV clinical manifestation transplant**						
Symptomatic	1.47	0.80–2.69	0.280			
Asymptomatic	**1.78**	**0.97–3.25**	**0.040**	2.08	0.47–11.11	0.060
**Reoccurring HCMV infection after HCMV prophylaxis**						
Donor positive	1.14	0.91–1.44	0.381			
Recipient positive	**2.14**	**1.12–4.09**	**0.011**	1.32	0.49–12.68	0.406
**Symptoms**						
**Fever**	0.75	0.40–1.38	0.362			
**Pneumonia**	0.82	0.43–1.56	0.562			
**Hematuria**	1.92	0.99–3.75	**0.032**	8.30	1.23–55.80	**0.029**
**Leukopenia**	2.25	0.97–5.20	**0.036**	3.52	0.52–23.52	0.193
**Diabetes**	2.70	1.19–6.09	**0.005**	9.33	1.24–70.01	**0.023**
**Diarrhea**	1.37	060–3.11	0.436			
**UTI**	2.01	0.94–4.22	**0.047**	2.14	0.33–13.59	0.419
**Biochemical parameters**						
**SGPT**	0.75	0.36–1.55	0.441			
**Alkaline phosphatase**	0.67	0.33–1.36	0.271			
**Creatinine level**	2.35	1.24–4.44	**<0.001**	9.43	1.35–65.59	**0.023**

**Table 4:** Quantitative comparison of selective risk factors among the HCMV infected renal rejected and non-rejected patients. The data were calculated using the mean ± SD followed by the use of Student’s *t*-test on the following groups.

		Less than Two Years after the Transplant (Early Rejection)				More than Two Years after the Transplant (Late Rejection)			
		Mean ± SD	*p*-Value	95% CI		Mean ± SD	*p*-Value	95% CI	
				Upper	Lower			Upper	Lower
**Antigenemia (no of cells/250,000 leukocytes)**	Renal Rejection	9.15 ± 2.41	0.030	−4.194	−0.225	1.71 ± 1.05	0.224	−0.850	0.208
	No Rejection	6.94 ± 2.98				1.39 ± 0.62			
**HCMV viral load (DNA log copies/µg)**	Renal Rejection	7.84 ± 0.89	0.007	−0.509	−0.343	2.96 ± 0.94	<0.001	−1.986	−0.987
	No Rejection	6.34 ± 1.08				1.47 ± 0.71			
**CD-4+ cell count**	Renal Rejection	515.68 ± 261.9	0.384	−279.20	110.34	460.36 ± 252.10	<0.001	121.94	411.48
	No Rejection	430.34 ± 261.3				827.24 ± 243.78			
**Creatinine level (mg/dL)**	Renal Rejection	1.57 ± 0.76	0.331	−0.756	−0.267	2.62 ± 0.81	<0.001	−1.794	−0.912
	No Rejection	1.33 ± 0.53				1.26 ± 0.67			

**Table 5:** Univariate comparison of risk factors and serum biochemical parameters between the HCMV-infected and non-infected renal rejected patient groups.

	Total Renal Rejected Patients (*n* = 55)		
	With HCMV Infection (*n* = 35) (%)	Without HCMV Infection (*n* = 20) (%)	*p*-Value
**Risk Factor**			
**Fever**	20 (57.14)	8 (40)	0.160
**Pneumonia**	16 (45.71)	7 (35)	0.476
**Hematuria**	23 (65.71)	5 (25)	**<0.001**
**Leukopenia**	24 (68.57)	6 (30)	**<0.001**
**Diabetes**	26 (74.28)	6 (30)	**<0.001**
**Diarrhea**	22 (62.85)	11 (55)	0.155
**UTI**	24 (68.57)	6 (30)	**<0.001**
**High Biochemical Parameters**			
**SGPT**	18 (51.42)	9 (45)	0.164
**Alkaline phosphate**	16 (45.71)	11 (55)	0.339
**Creatinine level**	20 (57.14)	13 (65)	0.547
**High CD-4+ cell count**	14 (47.17)	28 (70)	**0.031**

## Data Availability

All of the experimental data are available from the corresponding author, and for any further inquiries, you are requested to contact the corresponding author.
